# Generation and Characterization of Human Induced Pluripotent Stem Cells

**Published:** 2009-07

**Authors:** M.V. Shutova, A.N. Bogomazova, M.A. Lagarkova, S.L. Kiselev

**Affiliations:** 1 Vavilov Institute of General Genetics, Russian Academy of Sciences

## Abstract

Cell biology is one of the most rapidly developing branches in modern biology. The most interesting stages in early embryonic development for cell biology are those when a large number of cells are pluripotent. Inner-cell mass of blastocyst can be cultivated in vitro, and these cells are called embryonic stem cells. They are able to differentiate into different types of cells and tissues. But the greatest interest for practical application is the return (reprogramming) of adult cells into the pluripotent state. In our study for the first time induced pluripotent cells were derived from human umbilical vein endothelial cells by genetic reprogramming. We showed that these cells are similar to embryonic stem cells in their morphology, function, and molecular level. We are the first to show that reprogramming sufficiently changes X-chromosome chromatin state, which is normally inactive in female endothelial cells, towards its activation, providing evidence that endothelial cells are reprogrammed at an epigenetic level.

Amulticellular organism develops from a single cell, a zygote, and becomes a complex of mutually supported tissue types during its individual development. The totipotent zygote cell and the terminally differentiated cell contain the same set of genetic information, but this information is achieved differently. Cellular programs of differentiation happen at the genetic and epigenetic levels. The zygote achieves a specified program and divides, and at a certain stage, cells begin to specialize. A blastocyst (about 3.5 days in mouse and 5.5 days in humans) has two types of cells and is a future embryo which has no physical connection with its mother’s organism. The inner cells will develop into the organism and all its tissues, and the outer layer of cells will develop into trophectogerm, which will interact with the mother’s organism. In vitro (in laboratory conditions) cultivated blastocyst inner-cell mass were called embryonic stem cells (ESC). In vitro ESC under appropriate conditions do not continue their program further; they stay in a pluripotent state for an unlimited time [1], but one can induce their controlled differentiation into the tissues of all three germ layers just by changing the culture’s conditions [2]. However, in the case of using these type of cells in therapy, the immunologic compatibility between the derived tissues and the recipient remains to be defined. Reprogramming individual somatic cells to a pluripotent state will be a perfect solution to this problem. For this purpose, the technologies of somatic cell nuclear transfer into the oocyte and fusion of the somatic cell with the pluripotent one were developed [3-5]. However, in 2006, S. Yamanaka [6] put forward a method of somatic cell genetic reprogramming to the pluripotent state. For the induction of the pluripotent state, genes which encode the transcriptional factors essential for early embryonal development and maintenance of the pluripotent state Oct3/4 and Sox2, the antiapoptopic transcriptional factor Klf4, and the transcriptional factor, which maintain the cell’s proliferation c-Myc, were used. These cells were called induced pluripotent stem (iPS) cells. In the last three years, this technology has been significantly improved and doesn’t require obligatory genetic modification [7-9]. But the number of human cell types that were successfully reprogrammed remained very restricted [10, 11].

Cells which are able to generate iPS cells should have some distinct features. First, they should be sensitive to total reprogramming. Second, they should be available. Third, they should not have any accumulated DNA damage, for example, UV or other environmental skin-cell damage. And finally, the number of cells should be sufficient, and the reprogramming effective enough to minimize all possible DNA damage during in vitro manipulations. Based on the above-mentioned criteria, our aims were to choose an optimal cell type and genetically reprogram these cells to optimize their further use.

We decided to take human umbilical vein cells for reprogramming. They can be easily obtained; have not accumulated any DNA damage; can be obtained in large amounts without cultivation, proliferate well in culture only in the presence of factors that maintain their growth; and, in conjunction with the development of umbilical vein banks, they can be stored for a long time. There is no data in the literature about endothelial-cell reprogramming. The reprogramming was achieved by the use of genetic constructs which were tested in earlier experiments [6]. Human umbilical vein endothelial cells (HUVEC) were transducted with retroviral vectors which contained cDNA of Oct3/4, Sox2, KLF4 and cMyc genes. The multiplicity of infection was five viral particles. Six days after infection, the endothelial-cell culture medium was changed to an ESC medium. It is worth noting that endothelial cells do not proliferate in an ESC culture medium; this significantly enabled the selection of iPS cells. Three weeks after viral infection, morphologically identical ESC clones were selected (Fig 1).

**Fig. 1.**
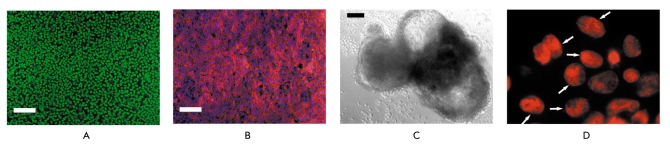
Morphology of iPS derived from an endothelium, feeder-free culture. A - Bright field image of iPS colony. B - Image of ESC colony. Bar scale - 100 mkm

The derived iPS cells did not differ in their the proliferative or morphological characteristics from human ESCs (Figs 2a, 2b). We confirmed with fingerprinting that these iPS were generated from human umbilical vein endothelial cells selected for reprogramming and were not the result of cellular culture contamination with human ES cells. To determine the number of integrated pro-virus copies, we used genomic hybridization with specific probes. Copy number varied from two to three copies of each virus per genome in derived iPS lines. These human iPS cells were pluripotent, formed embryonic bodies (Fig 2c), and differentiated into derivatives of all three germ layers. A karyotype analysis demonstrated that reprogrammed cells have a normal karyotype and retain it for at least 22 passages. iPS cells were cultivated in feederfree conditions in a mTeSR1-defined medium. Thus, we are the first to obtain iPS cells from human endothelium free of animal-derived components. The developed method allows us to obtain clinically applicable iPS cell lines.

**Fig. 2.**
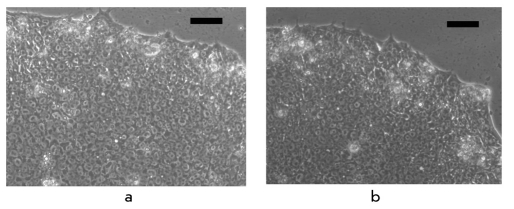
Feature analysis of human iPS cells derived from human umbilical vein endothelial cells. A, B - immunohistochemical analysis of iPS cells, antibodies stain to specific markers of pluripotency Oct4 (A) and SSEA-4 (B). The specific signals are stained with green (A) and red (B), nuclei in (B) are stained with blue (DAPI). C - embryonic bodies, derived from iPS cultured in suspension. Bar scale - 100 mkm. D - interphase nucleus in iPS cells stained with active chromatin marker H3me2K4 (red). White arrows localize the chromosome region of partly reactivated X - chromosome

Apart from changes in the realization of the genetic, program significant changes in the epigenetic state of somatic cells should take place during reprogramming. Inactivation of one of the two X-chromosomes in female cells occurs during early emryogenesis, although both X-chromosomes can be active in ESCs. Consequently, one can expect that during reprogramming there would be functional changes resulting in the reactivation of the inactive X-chromosome in endothelial cells. We used antibodies to active (H3me2K4) and nonactive (H3me3K27) chromatin to conduct an immunocytochemical analysis of X-chromosomes in derived iPS cell lines. Our data revealed that the marker of active chromatin (H3me2K4) appears on the nonactive X-chromosome in iPS cells (Fig 2d); at the same time, inactive chromosome in endothelial cells lacks an expression of this marker. Therefore, we are the first to show that, during genetic reprogramming, the reactivation of the inactive X-chromosome occurs in human cells. Our findings show that human endothelial cells can be effectively and completely reprogrammed to the pluripotent state, which was confirmed by morphological, molecular, and functional tests.

It is obvious that further experiments should be carried out for iPS cells application: for example, to confirm the reprogrammed state on the genome level and to confirm the oncogenic safety of iPS cells. This kind of research is one of the most promising avenues in the sphere of cell technologies. However, one should not forget that iPS cells are only an artificial analogue of ESCs: therefore, in order to identify their significance for application, two groups of pluripotent cells should be studied.

## Acknowledgements

This research was supported by the program of the Russian Academy of Sciences Biodiversity, grant RFBR 09-04-12199 ofi-m and OOO LKT (Moscow).
